# DNA Methylation Patterns of Chronic Explosive Breaching in U.S. Military Warfighters

**DOI:** 10.3389/fneur.2020.01010

**Published:** 2020-10-23

**Authors:** Zhaoyu Wang, Caroline M. Wilson, Yongchao Ge, Jeffrey Nemes, Christina LaValle, Angela Boutté, Walter Carr, Gary Kamimori, Fatemeh Haghighi

**Affiliations:** ^1^James J. Peters VA Medical Center, Medical Epigenetics, Bronx, NY, United States; ^2^Icahn School of Medicine at Mount Sinai, Nash Family Department of Neuroscience, New York, NY, United States; ^3^Department of Neurology, Icahn School of Medicine at Mount Sinai, New York, NY, United States; ^4^Center for Military Psychiatry and Neuroscience, Walter Reed Army Institute of Research, Silver Spring, MD, United States; ^5^Oak Ridge Institute for Science and Education, Oak Ridge, TN, United States

**Keywords:** blast exposure, breacher, epigenetics, DNA methylation, tinnitus, sleep, pain

## Abstract

**Background:** Injuries from exposure to explosions rose dramatically during the Iraq and Afghanistan wars, which motivated investigation of blast-related neurotrauma. We have undertaken human studies involving military “breachers” —exposed to controlled, low-level blast during a 3-days explosive breaching course.

**Methods:** We screened epigenetic profiles in peripheral blood samples from 59 subjects (in two separate U.S. Military training sessions) using Infinium MethylationEPIC BeadChips. Participants had varying numbers of exposures to blast over their military careers (empirically defined as high ≥ 40, and conversely, low < 39 breaching exposures). Daily self-reported physiological symptoms were recorded. Tinnitus, memory problems, headaches, and sleep disturbances are most frequently reported.

**Results:** We identified 14 significantly differentially methylated regions (DMRs) within genes associated with cumulative blast exposure in participants with high relative to low cumulative blast exposure. Notably*, NTSR1* and *SPON1* were significantly differentially methylated in high relative to low blast exposed groups, suggesting that sleep dysregulation may be altered in response to chronic cumulative blast exposure. In comparing lifetime blast exposure at baseline (prior to exposure in current training), and top associated symptoms, we identified significant DMRs associated with tinnitus, sleep difficulties, and headache. Notably, we identified *KCNN3, SOD3, MUC4, GALR1*, and *WDR45B*, which are implicated in auditory function, as differentially methylated associated with self-reported tinnitus. These findings suggest neurobiological mechanisms behind auditory injuries in our military warfighters and are particularly relevant given tinnitus is not only a primary disability among veterans, but has also been demonstrated in active duty medical records for populations exposed to blast in training. Additionally, we found that differentially methylated regions associated with the genes *CCDC68* and *COMT* track with sleep difficulties, and those within *FMOD* and *TNXB* track with pain and headache.

**Conclusion:** Sleep disturbances, as well as tinnitus and chronic pain, are widely reported in U.S. military service members and veterans. As we have previously demonstrated, DNA methylation encapsulates lifetime exposure to blast. The current data support previous findings and recapitulate transcriptional regulatory alterations in genes involved in sleep, auditory function, and pain. These data uncovered novel epigenetic and transcriptional regulatory mechanism underlying the etiological basis of these symptoms.

## Introduction

Injuries from exposure to explosive blasts rose dramatically during Operation Iraqi Freedom and Operation Enduring Freedom (OIF, OEF) due to the increased use of improvised explosive devices (IEDs) in military settings and in civilian populations through acts of terrorism (1, 2), which have motivated investigations of blast-related neurotrauma. Despite this increase in occurrences, our understanding of the effects of blast and the mechanisms behind subsequent brain injury remains limited (3, 4). Toward this effort, the John S. McCain National Defense Authorization Act (NDAA) for Fiscal Year 2019 (5) passed by Congress emphasized the importance of preventing blast-related traumatic brain injury (TBI) in both combat and training sessions. The bill called for a review of the cognitive effects of blast exposure including both the effects of successive blast events, and the feasibility of understanding the cumulative (lifetime or annual) limits of blast exposure (2). Further, in recognition of the latent residual effects of exposures to blast, the 2020 NDAA passed by U.S. Congress also ordered the history of blast exposure and blast duration from both combat exposure and trainings to be included in medical histories of Service Members in order to inform future risk mitigation and determination of injury and related sequelae (6).

In an effort to understand the acute and chronic physiological and cognitive effects of blast exposure in military personnel, we have focused on military Breachers, a unique population who are, by occupational definition, typically in close proximity to controlled, low-level blast during explosive breaching operations and training, and repeatedly exposed to primary blast overpressure waves. Breachers may apply explosives as a means of gaining access to barricaded or hardened structures, where they can be exposed to as many as a dozen 0.3–10 lb charges per day during training exercises and larger numbers and magnitudes per day during military operations.

Exposure to blast often leads to polytrauma (multiple traumatic injuries) and a multisystem response [for an in-depth review see (7)]. Shock waves from explosive blasts can damage both gas- and fluid-filled structures of the body, including the lungs, intestines, brain, eyes, nose, and middle ear (7–11). Specifically, damage to the auditory system can be the consequence of either direct exposure of the auditory canal to blast shock waves or TBI and neurological impairment affecting central auditory processing involving different brain regions after blast exposure (12). It can be difficult to determine which neurologic symptoms are linked to blast-related TBI separate from those that may be related to other kinds of injury from the blast that affects the nervous system secondarily. Depending on blast injury severity, neuropathological and clinical symptoms can include neuronal swelling, subdural hematomas, myelin deformation, inflammation, loss of consciousness, temporary disorientation, sleep disturbances, memory deficits, and tinnitus (ringing in the ear) (12–20).

Based on concerns for potential injury by the cadre of breachers and instructors, the Department of Defense (DOD) has been conducting studies on the bio-effects from repeated exposure, and findings from our own group (21) and others (22, 23) have begun to show that blast exposure during training is capable of inducing changes in DNA methylation and gene expression in military breachers (22) that track with the physiological symptoms of blast injury (24). In the present study of military breachers, we set out to replicate previous findings, investigating effects of acute and chronic blast exposure on DNA methylation and reported symptoms at baseline across two military training sites–representing the largest DNA methylation study of breachers to date. In previous work, we found no significant DNA methylation changes associated with acute blast exposure (3-days post blast) (21). In the present study, we investigated whether blast induced DNA methylation changes could be detected at a more proximal time point, within 2-h post-blast exposure. Additionally, in line with our ongoing work in understanding the chronic biosignatures of accumulative blast exposure, we investigated whether DNA methylation from peripheral blood captures chronic cumulative exposures to blast and associated symptoms in this independent cohort of breachers. To study the chronic effects of blast exposure and associated biological mechanisms, we examined baseline (i.e., before training) transcriptional regulatory profiles in operational blast training, comparing participants with low vs. high cumulative lifetime blast exposures. We further examined the high vs. low cumulative exposed groups' reported physiological and psychological symptoms and aim to identify DNA methylation changes that associate with frequently reported symptoms by breacher training participants, which included sleep disturbance, tinnitus, and headache.

## Methods

### Samples Demographics and Symptoms

All subjects consented to participate in the study and the human use protocol for interaction with the subjects was approved by Institutional Review Board (IRB) of the Walter Reed Army Institute of Research (Silver Spring, MD) and chains of command prior to data collection. The procedures were followed in accordance with the ethical standards of the IRB, Army Regulation 70-25, and the Helsinki Declaration.

Data were collected over 3-days at two training sites, situated at Fort Leonard Wood, MO, from 59 male subjects. Demographic information including sex, age, lifetime operational exposure to blast, and self-reported lifetime TBI history were recorded at the start of the training (see Supplementary S1). Also, self-report symptom assessments were completed at each time point that blood was collected. The self-report symptom assessment included a range of symptoms related to blast injury, including tinnitus, sleep difficulties, and headache, as well as additional symptoms based on the Rivermead Post-Concussion Symptom Questionnaire (RPSQ) (25) and surveys derived from aggregations of concussion symptomology present in current clinical and research findings, along with relevant Veterans Affairs and CDC Annual Report materials (24, 26, 27). The symptom assessment was developed to be administered on a single page and for minimal disruption of, or interference with, the operational duty of the personnel whose responsibility first and foremost is training. This is due to the fact that it is not feasible to incorporate administration of detailed and time-consuming clinician administered assessments that would interfere with operational duties and responsibilities of the participants. Despite its brevity, the assessment is an inclusive list of blast symptomology, with the benefit of being neutrally worded to ameliorate potential underreporting by participating military service members (24). The neutrality of this survey is crucial given the possibility of underreporting military operation-associated symptomology by service members, particularly for symptoms associated with mental health status, given the potential stigma around possible fitness for operational duties (24, 28).

### DNA Methylation Sample Processing and Quality Control (QC)

Whole blood was collected using PAXgene blood DNA tubes (PreAnalytix), and stored at −80°C. Genomic DNA was isolated using PAXgene Blood DNA kit (PreAnalytix). Genomic DNA was bisulfite converted (Zymo Research) and CpG methylation determined using Illumina Infinium HumanMethylation EPIC BeadChip microarrays, as described previously (29). Data and QC analyses was performed using R Language 3.4.2 (30), an environment for statistical computing, and Bioconductor 2.13 (31), and all raw data files (.idat) processed by the minfi package (32). All samples were subjected to quality control procedures for sample tracking and sex prediction analyses as follows. All samples but one displayed >99% of probes that passed detection call < 0.00005 (Figure S1), and the one sample did not pass was dropped and was not included in downstream analyses. Sex QC analysis also confirmed methylation-based sex prediction with those reported (all male, Figure S2). It should be noted that a single reference female sample was included for the sex QC analysis (Figure S2). For QC sample tracking of pre- vs. post-blast exposure breacher training, we used the 59 single nucleotide polymorphism (SNP) probes included in the Human MethylationEPIC BeadChip, confirming that the subjects from FLWB training site for which multiple samples at multiple time points were assayed grouped together (Figure S3).

### DNA Methylation Data Analysis

For all DNA methylation analyses, we used the matrix of M-values (logit transformation of beta-values) which correspond to methylation levels. Surrogate variable analysis (SVA) was performed to add surrogate variables and rule out potential batch effects. A linear model was used for the binary variable of interest, while including age and history of TBI as covariates in the model. Performing the comparative analysis in limma (33) implemented in *R*, we obtained *t*-statistics and associated *p*-values for each CpG site. The point-wise *p*-values, were then used for the identification of differentially methylated regions (DMRs) using the combined-*p*-value tool (34), and all DMRs identified by the combined-*p* value tool are all significant. For baseline low vs. high cumulative blast symptom DNA methylation analyses, we used the same matrix of *M*-values as above, where for each symptom, a vector of symptom scores at day 1 is added to the design matrix as a new variable to perform linear regression in limma, with point-wise *p*-value and significant CpG sites done in the same manner. Moreover, for the pre-post blast symptom analysis, we used the matrix of change in *M*-values as in pre-post methylation analyses and dichotomized the symptom variable where for each selected/filtered symptom we compared symptom score pre-post; if the score increased, the variable was set to equal one, otherwise it was set to zero.

Furthermore, due to the potential contribution of cellular heterogeneity of blood sample specimens and its effect on DNA methylation patterns, we examined whether the variability in cell proportions may be a potential confound between methylation and our factors of interest. Therefore, we used Horvath's DNA methylation age calculator (35), a tool to estimate DNA methylation age and cell proportions for samples on the Illumina Infinium platform (36) in order to calculate the cell proportion estimates of six cell types (CD4 T-cell, CD8 T-cell, natural killer, B-cell, monocytes, and granulocytes), and compared each cell's proportions between groups in both studies (low vs. high cumulative blast exposure and acute pre- vs. post-blast exposure). None of the cell types showed different cell proportions within the low-high study, while CD4T, natural killer and B-cell showed significant differences between the pre- and post-exposure samples; and thus their proportion differences (post- minus pre-) were added as covariates in subsequent DNA methylation analyses. Specifically, for the pre- and post-blast exposure analysis, a matrix of difference *M*-values (post- minus pre-blast) was used in the linear model, including the additional covariates from the cell proportion estimates. Lastly, for replication of findings across independent data sets, DMRs were compared such that if the directionality of gain or loss of DNA methylation within the differentially methylated region(s) and associated CpG sites were consistent across both datasets, it was considered a replication.

## Results

In the present study we examined genome-scale DNA methylation patterns using the Illumina Infinium HumanMethylation EPIC BeadChip at baseline (pre-breacher training) amongst two cohorts of military trainees in 3-days period during an explosive breaching course at Fort Leonard Wood (FLW). This consisted of 28 participants from one training session (FLWA) and 31 participants from second, separate session (FLWB), totaling 59 sample subjects (for demographic breakdown of each subject per site and respective breaching history, see Table S1). In an attempt to replicate our previous findings, for one of these sites (FLWB), we also investigated DNA methylation changes following exposure to operational blast exercises, which corresponded to 29 participants with available biospecimens that passed QC [see Methods DNA methylation sample processing and Quality Control (QC)].

### DNA Methylation Changes Are Not Significantly Associated With Acute Blast Exposure

**Figure 1:**
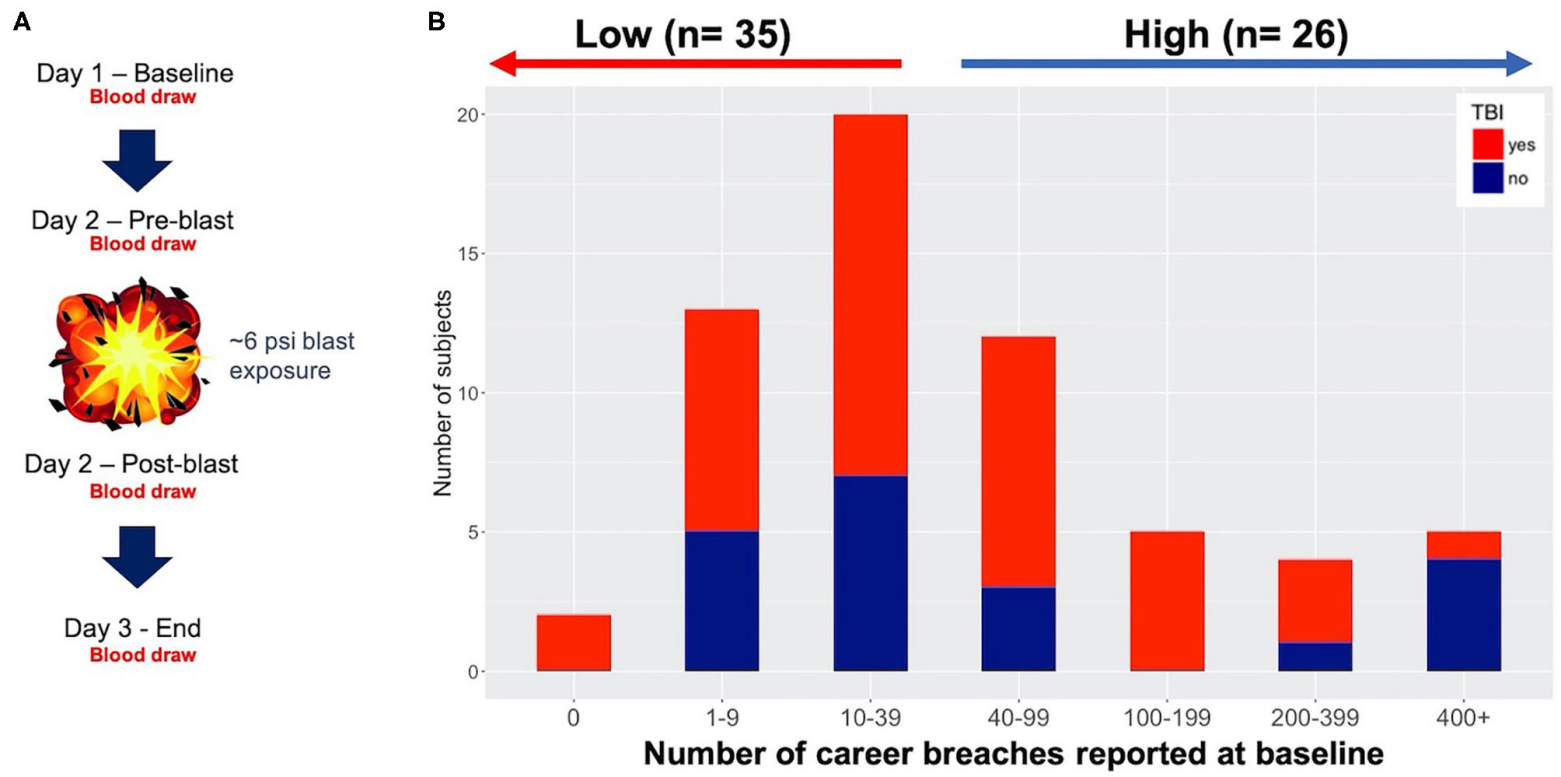
Study design and participants. **(A)** 3-day protocol showing blast exposure and blood draws throughout the study. In this study we focus on baseline data only using specimens from the pre-blast timepoint. **(B)** Number of career breaches reported at baseline (61 participants) with and without history of traumatic brain injury (TBI) (red and blue, respectively), with ≤ 39 blasts empirically defined as low blast, and 40 ≤ career breaches considered high exposure to blast.

We previously reported that DNA methylation changes do not track with acute exposure to blast (21), and our present findings further support this in that DNA methylation appears to not be altered acutely pre- vs. post-blast exposure. As noted, for this experiment we used biospecimens from one site, FLWB, and examined DNA methylation changes in day-2 of training, pre vs. post blast exposure (approximately within 2 h post blast exposure, see Figure 1A for training protocol). DNA methylation assays were conducted from whole blood samples. When accounting for intra-individual differences in cell proportions (as described in Methods DNA methylation data analysis), we were unable to detect significant changes in DNA methylation before and after exposure to blast (see Table S2).

### Lifetime Cumulative Blast Exposure Alters DNA Methylation in High Relative to Low Blast-Exposed Groups

We have previously shown that lifetime cumulative blast exposure is capable of altering DNA methylation in our investigations of military breachers with high relative to low lifetime blast exposures at baseline among military service members participating in a 10-days explosive breaching training course (21). Here, we also investigate at baseline, alterations in DNA methylation in 59 military trainees from two independent cohorts over a 3-days period during the explosive breaching course at Fort Leonard Wood (FLWA and FLWB sites, Figure 1A). The history of number of self-reported career breaches–that is, number of breaching exposures throughout a career in military service–were recorded at baseline for the two separate 3-days training cohorts reported here (59 participants total), as well as self-reported history of TBI (shown together in Figure 1B and Table S1). However, we first determined whether participants from these two separate training sessions were comparable to be combined for subsequent analyses in this study. In particular, we found no significant difference between the two sites based on the participant's age (*t*-test, *p* = 0.3666), lifetime history of mild TBI (χ^2^, *p* = 0.1424), or lifetime career breaching history (χ^2^, *p* = 0.8214). As such, all data including DNA methylation data for the two sites were combined for subsequent downstream analyses. For the comparative analyses, we considered a total of 33 participants with reported low number of lifetime career breaches (≤ 40), and 26 trainees with reported high number of career breaches, ranging from 40 to more than 400 breaches (Figure 1B). The criterion for low vs. high cumulative career breaching experience used here was empirically defined, and was based on our previous work (21). It should be noted that estimation of cell proportions in whole-blood specimens, showed no significant differences by cell types within the low vs. high groups (data not shown), and thus were not adjusted for in the analytical models going forward. Also, there was no correlation between history of TBI and the total number of lifetime career breaches (χ^2^ = 9.15, *p* = 0.1655).

**Figure 2:**
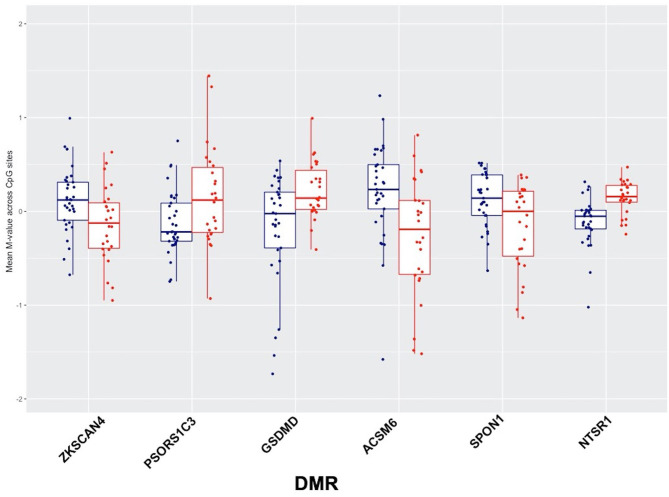
Six significantly differentially methylated regions (DMRs) identified using the combined *p*-value tool in high relative to low-blast exposed groups, showing low blast exposed groups in blue and high blast exposed groups in red, with mean methylation (represented by *M*-values) across CpG sites for each subject on the y-axis.

Comparing DNA methylation changes in high relative to low cumulative blast exposure groups at baseline, we identified six significantly differentially methylated regions (DMRs) that passed rigorous multiple testing corrections (Table S3), with four DMRs overlapping with regulatory and protein coding regions, (i.e., promoter and genic features, shown in Figure 2). Notably, DMRs overlapping with loci associated with autoimmune disorders (37–39), *ZKSCAN4* (zinc finger with *KRAB* and *SCAN* domains 4) and *PSORS1C3* (psoriasis susceptibility 1 candidate 3), localized to the major histocompatibility complex region on chromosome 6 show a loss of methylation in the *ZKSCAN4* DMR and a gain of methylation in the *PSORS1C3* DMR in the group with high cumulative exposure to blast (Figure 2). Additionally, DMRs overlapping with the loci *SPON1* (spondin-1), with loss of methylation, and NTSR1 (neurotensin receptor 1), with gain of methylation, were also identified in subjects with high cumulative exposure to blast (Figure 2). Both *NTSR1* and *SPON1* are implicated in circadian rhythm cycles and dysregulated sleep.

### Symptoms Associated With Cumulative Blast Exposure and Associated Differentially Methylated Regions in High Relative to Low Blast-Exposed Groups

**Figure 3:**
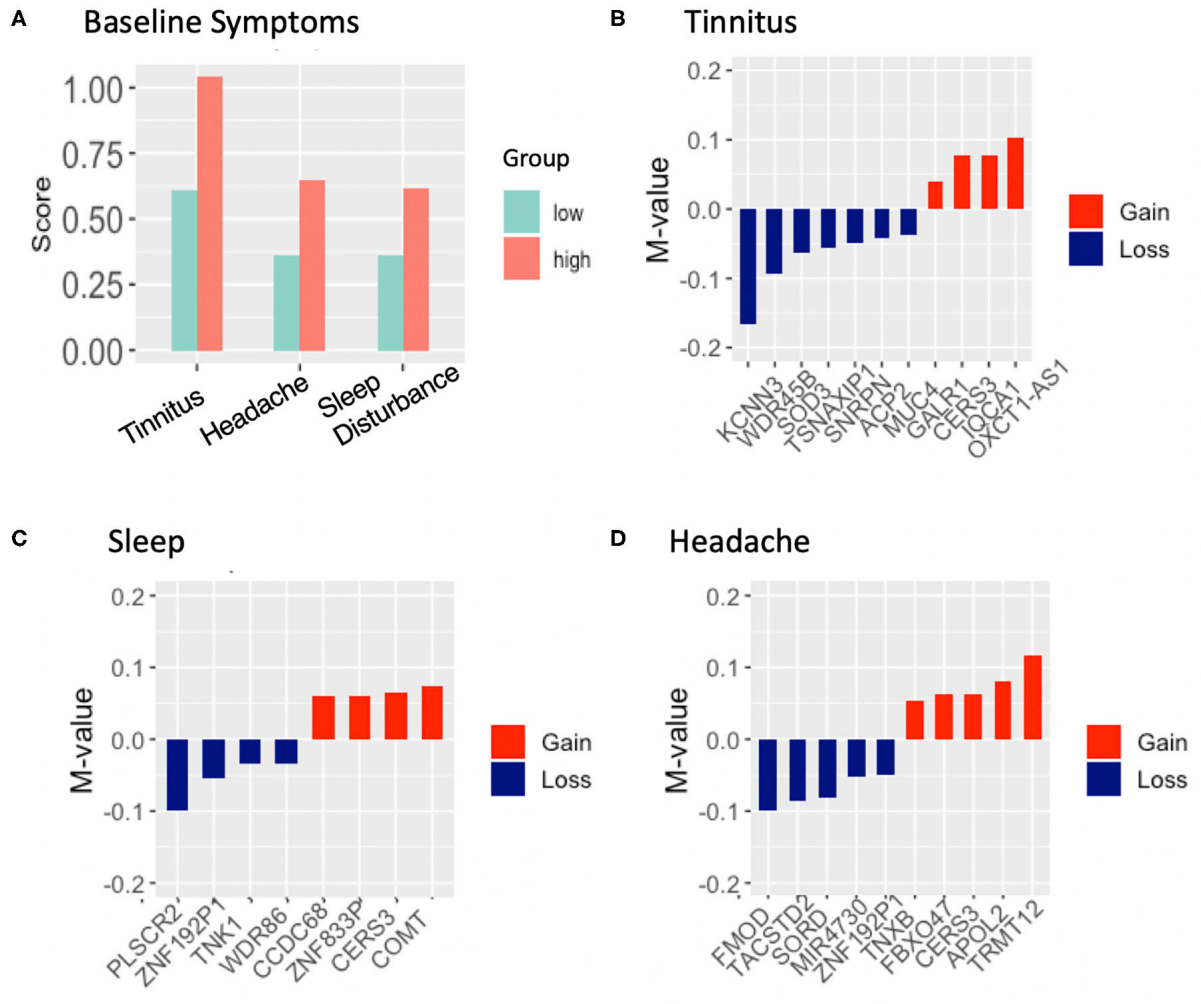
**(A)**, Frequency of endorsed symptoms at baseline in 61 subjects across high and low blast exposed groups. Tinnitus (ringing in the ear) was the highest reported symptom, endorsed by 29 participants; **(B–D)**, differentially methylated regions and associated genes that track with the reported symptom of tinnitus, headache, sleep difficulties in high relative to low lifetime blast exposed groups, showing gain of methylation in red and loss of methylation in blue, with mean methylation (represented as *M*-values) across CpG sites (between the high group relative to the low blast exposed group) shown on the y-axis.

Self-reported neurological and physiological symptoms endorsed by participants in the breacher cohorts were ascertained at the start of the training (prior to blast exposure) and we utilized this information to track DNA methylation changes that co-occured with symptoms associated with cumulative exposure to blast. We previously demonstrated that symptoms in high relative to low blast exposed groups tracked with DNA methylation patterns (21), where tinnitus (ringing in the ear) was significantly associated with changes in 18 differentially methylated regions. In the present study, tinnitus was the top reported symptom at baseline, with 49% of participants reporting tinnitus at “baseline” (assessed pre-blast in day-2 of training depicted in Figure 1A protocol, with symptom frequencies shown in Figure 3A). The subsequent top symptoms (as also shown in Figure 3A) were forgetfulness (34% reported), headache (35% reported), and sleep disturbances (25% reported). No significant DMRs were identified for the reported symptom of forgetfulness that tracked with cumulative blast.

DNA methylation analyses at baseline, in high relative to low cumulative blast exposure groups with the reported symptom of tinnitus identified 14 DMRs (Table S4), with 11 of these DMRs overlapping with promoter and genic features of protein coding genes (or experimentally validated loci Figure 3B). Remarkably, we found that five out of 11 (45%) of these DMRs were associated with genes involved in auditory functioning or hearing loss through this unbiased genome wide approach. These include the genes KCNN3 (potassium calcium-activated channel subfamily *N* member 3), MUC4 (mucin 4, cell surface associated), SOD3 (superoxide dismutase 3), WDR45B (WD repeat domain 45B), which show a loss in methylation in the high cumulative exposed group, and GALR1 (galanin receptor 1), which show a gain in methylation. Additionally, we observed 10 DMRs that tracked with the reported symptom of sleep difficulty in high relative to low lifetime blast exposed participants (Table S4), with 8-DMRs overlapping with genic and promoter regulatory regions (Figure 3C). Notably, two loci COMT (catechol-O-methyltransferase) and CCDC68 (coiled-coil domain containing 68) have previously been associated with sleep disturbance and insomnia. Both show a gain of methylation that track with sleep disturbance in those with high accumulative blast exposure. Finally, headache, the third symptom that passed filtering and tracked with DNA methylation patterns in high vs. low blast exposed groups, yielded 13 DMRs (Table S4), and of these, 10-DMRs overlapped with genes and promoter regulatory regions (Figure 3D). Although no previous studies have linked these loci to headache symptoms, we did identify two loci FMOD (fibromodulin), which shows a loss in methylation, and conversely TNXB (tenascin XB), which shows a gain in methylation in high blast exposed groups that have been previously investigated in studies involving temporomandibular joint dysfunction (TMJ) and chronic pain (40).

## Discussion

We identified changes in DNA methylation and gene expression in a 10-days explosive breaching training course (21), emphasizing the effects of cumulative blast on sustained DNA methylation alterations, specifically related to chronic symptoms of sleep disturbances and tinnitus in our previous work. Here, we examined DNA methylation data from 59 military trainees with varying lifetime histories of exposure to blast during a 3-days training session, in conjunction with symptom and demographic information, in order to determine novel DNA methylation signatures in association with blast exposure and blast-related symptoms, and importantly attempt to replicate our previous findings in studies of breachers (21). In line with our previous findings, we showed that DNA methylation changes do not encapsulate physiological changes acutely, pre-post blast exposure, suggesting other molecular mechanisms for symptoms experienced acutely following exposure. However, also as shown previously (21), we found that DNA methylation signatures appear to encapsulate long-term chronic exposure to blast.

### DNA Methylation Alterations Associated With Cumulative Exposure to Blast

Similar to our previous work, in comparing DNA methylation changes in high relative to low cumulative blast exposure groups at baseline (21), we identified six significant DMRs that passed multiple testing corrections, wherein *SPON1* and *NTSR1* were identified as significantly differentially methylated in the low vs. high blast exposure analysis (Figure 2). Both *NTSR1* and *SPON1* are implicated in circadian rhythm cycles (41, 42), further confirming that chronic exposure to blast may dysregulate sleep. A study of intrinsically photosensitive retinal ganglion cells in the suprachiasmatic nucleus identified *F-spondin* as significantly enriched in the suprachiasmatic nucleus (SCN). *F-spondin* deficient mutants (*spon*^−/−^) demonstrated severely disrupted “free running” rhythmicity, suggesting a novel role for F-spondin in maintaining intrinsic circadian rhythm cycles (42). Our low vs. high career breaching DNA methylation analyses also identified *NTSR1* as significantly differentially methylated. In animal studies of NTSR1, NTSR1 knockout mice had a lower percentage of time spent in REM sleep relative to wild-type (41). Furthermore, following sleep deprivation, *NTSR1* knockout mice (C57BL/6N wild-type mice with a targeted *Ntsr1* mutation) exhibited more wake and less NREM rebound sleep, and also showed increased anxiety and despair behaviors (41).

**Figure 4:**
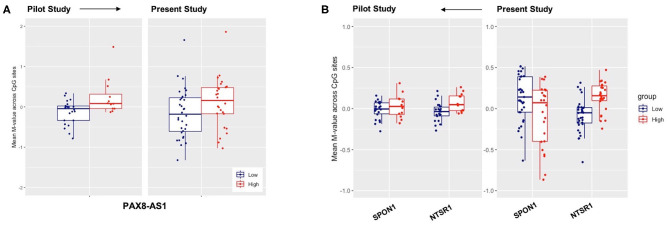
Replication of previously reported genes for low-high blast exposure analyses of genes **(A)** *PAX-AS1* and **(B)** *SPON1* and *NTSR1*, with mean methylation *M*-values across CpG sites on the y-axis and the respective mean *M*-value across CpG sites for both low-(blue) and high (red) lifetime blast exposed individuals.

To confirm previous findings, we examined whether data from our prior and current studies replicate. Comparing DNA methylation data between our previous findings (21) and the present study using an independent cohort, we investigated DNA methylation patterns in *PAX8-AS1*, an antisense transcript of *PAX8*, a transcription factor associated with thyroid function. Genome-wide association studies have implicated variants associated with *PAX8* and sleep duration (43–45). We observed similar trends in terms of gain of methylation associated with high levels of lifetime blast exposure in this study for the *PAX8-AS1* gene, such that mean methylation levels in *PAX8-AS1* were higher in breachers with high lifetime cumulative blast exposure in both previous and present findings (Figure 4A). However, the antisense RNA, *PAX8-AS1*, was not differentially methylated in low vs. high cumulative blast groups following correction for multiple testing.

We also investigated DNA methylation patterns of *NTSR1* and *SPON1* genes across the two breaching protocols in the current FLW and previously published study. We found that the methylation pattern of the *NTSR1* DMR replicated across both studies such that breachers with high lifetime exposures to blast had higher mean-methylation of *NTSR1* (Figure 4B). Yet, we did not observe the same directionality in gain of methylation in the *SPON1* DMR in the present study relative to our previously published data (Figure 4B). However, it is possible that the methylation change is not high enough to be detected across all CpG sites. Our findings on sleep related genes in the breacher populations are highly translational, given the high prevalence of insomnia in service members and veterans (46, 47). Amongst a cohort of Veterans who had recently incurred combat-related mild TBIs (both blast and blunt trauma-induced), nearly all endorsed sleep issues, but those with specifically blast-induced mild TBI developed higher rates of anxiety and insomnia than those with blunt injuries (48). Taken together, these data suggest that the mechanism of blast injury may differently influence underlying molecular interactions, wherein recognition of the mode of injury may be crucially important for development of targeted clinical treatment and rehabilitation.

### DNA Methylation Tracks With Chronic Exposure to Blast and Related Symptoms

We identified changes in DNA methylation that tracked with top self-reported symptoms of tinnitus, sleep difficulties, and headache (Figure 3A).

#### Tinnitus

Notably, tinnitus symptom analyses identified regions with differential methylation in genes *KCNN3, MUC4, GALR1, SOD3*, and *WDR45B* (in the low vs. high cumulative blast exposed groups (Figure 3B). Again, to confirm our previous findings, we examined whether the tinnitus findings from our prior and current studies replicate. We first investigated DNA methylation patterns in *KCNN3, MUC4, GALR1, SOD3*, and *WDR45B* genes that are implicated in auditory functioning and hearing loss across the two breaching protocols in the current FLW and previously published studies, and found that the methylation patterns of the DMRs in these loci did not replicate. However, comparing DNA methylation data from our previous findings (21) with the current FLW dataset, we observed that the genes Cytochrome P450 Family 2 Subfamily *E* Member 1 (*CYP2E*1**)** showed the same pattern in gain of methylation, and the gene dual specificity phosphatase 22 (*DUSP22*) showed the same pattern in loss of methylation, amongst breachers with high cumulative exposure to blast. Relevant to the symptoms of tinnitus, deletions of the *DUSP22* gene in humans results in severe intellectual disability and deafness (49, 50), while *CYP2E1* is integral to the metabolism of acrylonitrile, which has been shown in rodents to potentiate damage to hair cells in the inner ear.

Importantly, we have identified novel genes differentially methylated that are associated with tinnitus symptoms in breachers with cumulative exposure to blast in the present study (Figure 3A). Specifically, potassium channel genes, along with *KCNNN3* and other genes in this family, play a critical role in defining the electrophysiological properties involved in the response patterns of auditory neurons (51, 52). The electrophysiological properties of auditory neurons are governed by the neuronal circuitry, cellular morphology, and patterns of potassium channel subunit expression. The cochlear nucleus is the only central nervous system region to receive direct innervation from the auditory nerve, and a recent systematic gene expression profiling study in rodents identified transcripts for 51 potassium channels (including *KCNN3*) within the cochlear nuclei subdivisions (i.e., the anterior ventral, posterior ventral, and dorsal), showing highest KCNN3 gene expression in the anterior ventral cochlear nuclei (53). Data from the present study provides the first association of *KCNN3* gene with the symptom of tinnitus in human samples.

To our knowledge, this study also provides the first human data supporting the association of *SOD3* with tinnitus symptoms in breachers with repeated blast exposure. In a rodent study, mice with repeated exposure to blasts showed injury to the auditory cortex and coordinated gene expression changes in genes known to be involved in age- or noise-induced hearing impairment across multiple brain regions, where increase *SOD3* expression in the hippocampus was observed following blast exposure (12). This increase in *SOD3* expression is potentially a protective response to blast injury, since superoxide dismutase are critical antioxidants that work against oxidative stress in the body (54). Indeed, the functional role of reactive oxygen species and the protective efficacy of antioxidants in noise-induced hearing loss are well-established (55–57). Repeated blast injury can induce production of reactive oxygen species (58) leading to oxidative stress, suggested as a possible mechanism for tinnitus, given that oxidative stress can impact hair cells, cochlear degeneration, and neural-auditory pathways (59–61).

Furthermore, *GALR1* and *WDR45* loci identified in the present study have been linked to congenital developmental auditory dysfunction. A large mutation screening of 307 deafness genes in patients with microtia identified *GALR1* as a strong candidate gene (amongst others) with novel mutations associated with microtia (62). Microtia is a malformation of the external ear that ranges in severity from mild differences in auricular shape and size to complete absence of the external ear with atresia or stenosis of the auditory canal that may be caused by genetic and/or environmental factors (63, 64). Microtia patients can suffer from range of symptoms including conductive hearing loss and sensorineural hearing loss (65). Interestingly, a case report of a patient with congenital aural atresia (typically resulting in unilateral or bilateral ear malformation) caused by chromosome 18q deletion, also contained the *GALR1* gene (66). Additionally, mutations in the *WDR45* loci have resulted in beta-propeller protein-associated neurodegeneration (BPAN), a rare form of neurodegeneration resulting from an accumulation of iron in the brain (67). Patients with WDR45 mutation also exhibit lateral sensory neural hearing loss and auditory agnosia (68) Similarly, in rodents *WDR45* knockout mice exhibit either an increased or absent auditory brainstem response when assessed using an 18 kHz-evoked Auditory Brain Stem Response Threshold test, compared to wildtype animals (69).

Lastly, the *MUC4* gene also was found to track with symptom of tinnitus in breachers, and has been previously implicated in mucoid otitis media (MOM) (70) a common otological disease that can result in hearing loss. MOM is a chronic condition that can persist long-term leading to conductive hearing loss (71). Pathologically, MOM is characterized by accumulation of mucous effusion in the inner ear that reflects high concentrations of mucins, including *MUC4*. Mucin hyperproduction involving *MUC4* overexpression have been observed under inflammatory conditions (72–74), which is in line with the inflammatory response also observed following acoustic injury (75), suggesting that immune responses may underlie biological and molecular processes associated with acoustic trauma. These findings are particularly relevant because blast exposure is reported to cause auditory impairment in a large population of service members (76, 77). Auditory/vestibular injuries from blast traumatic brain injury (TBI) can cause increased incidence of tinnitus and hearing loss, which when left untreated can worsen over time (76–79). In fact, tinnitus is the most prevalent service-connected disability of all Veterans Benefit Administration compensation recipients (80), in addition to being the most commonly reported symptom by breachers in former and this present study.

#### Sleep Disturbance

In addition to DMRs that tracked with the symptom of tinnitus, we also identified DMRs that tracked with the symptom of sleep difficulties (Figure 3C). Interestingly, the gene CCDC68, coiled-coil domain containing 68, has been shown to be significantly associated with the sleep disturbance phenotype in genome wide association studies (81–83). Moreover, *PLSCR2*, phospholipid scramblase 2, and TNK1, tyrosine kinase non-receptor 1, are involved in phospholipid metabolism, which is significant because lipid signaling and is associated with both sleep and synaptic function (84). *ACSF3*, an acyl co-A synthetase, was also identified as differentially methylated and plays a role in fatty acid synthesis (85), which is a critical process that sustains brain energy metabolism during sleep (86). Interestingly, we also observed *COMT* as differentially methylated and significantly associated with the reported symptom of sleep disturbance, and COMT has been implicated in and circadian physiology (87–89). The catechol-*O*-methyltransferase enzyme modulates dopamine levels within the prefrontal cortex (PFC), where it metabolizes dopamine and renders it inactive (87, 88). A single nucleotide polymorphism within the *COMT* gene With a valine (Val) to methionine (Met) substitution at codon 158 (Val158Met) (90) differentially affect COMT's enzymatic activity and thus dopamine levels within the PFC. Specifically, the Met allele results in a ~4-fold reduction of the enzymatic activity compared to the Val allele, leading to increased dopamine within the PFC (87, 90, 91). In a study involving subjects with sleep deprivation, those with the Val allele showed greater impairment in adaptive decision making as compared to those with the Met allele (92). Using actigraphy data another study showed that Val/Val and Met/Met homozygotes habitually prolonged sleep on rest days compared to workdays, whereas Val/Met heterozygotes did not significantly increase their sleep duration (93) suggesting that the Val/Met polymorphism may be associated with inter-individual differences in distinct aspects of sleep-wake regulation and physiology. The association between chronic lifetime cumulative blast exposure and changes in DNA methylation in these loci previously implicated in sleep and circadian function is highly relevant, because sleep disturbances are commonly reported in military service members within both active duty and post-deployment settings (94–96).

#### Headache and Chronic Pain

A number of genes were also identified that tracked with the reported symptom of headache (Figure 3D). In particular, the gene *FMOD* (fibromodulin) a collagen-binding molecule expressed in connective tissues is particularly interesting given its role in pathobiology in osteoarthritis of the temporomandibular joint (40, 97). A number of rodent models involving *FMOD* gene knockouts have shown accelerated osteoarthritis in the temporomandibular joint (40), as compared to wildtype animals. Temporomandibular joint disorder has been reported by service members and veterans with exposure to IEDs and blast injury (98) and posttraumatic stress disorder (PTSD) (99). Of relevance to the data presented, headaches are the most commonly reported condition associated with temporomandibular joint disorders (100). Veterans with blast mTBI report high prevalence of chronic daily headaches and migraines, as compared to the general population with TBI and concussion (101–103). Another gene *TNXB* (tenascin XB) was also found in the present study to track with headache in breachers with high cumulative blast exposure. Mutations in the *TNXB* locus are associated with joint hypermobility syndrome, which is a connective tissue disorder characterized by chronic musculoskeletal pain due to joint hyperextensibility (104). This is considered a milder form of Ehlers-Danlos syndrome, in which *TNXB* locus is also implicated (104, 105). These disorders have highly variable clinical presentations, which include chronic pain and headaches (104). Headaches can be the primary complaint or refractory due to tension, temporomandibular joint dysfunction, or unilateral myofascial pain etc. (104, 106). The two key loci *FMOD* and *TNXB* identified here that track with headache and chronic pain may provide insight to the etiological basis of chronic pain reported by our service members and veterans with blast related mild TBI (107, 108).

### Study Limitations

This study has a number of limitations. First, this study lacks representation of both sexes, for all our participants were male; therefore, our findings may not be generalizable to the small (but growing) population of female service members and veterans. The growing increase in female population in the United States Armed Forces and recent inclusion of women in combat roles investigation of sex differences can be the focus of future studies. Additionally, as noted in Methods samples demographics and symptoms, the self-report symptom assessment used was based on the RPSQ and surveys derived from aggregations of symptoms associated with clinical concussion symptomology, supported by clinical and research findings (24, 26, 27) and these measures likely include a number of separable factors [e.g., (109)]. Therefore, a larger sample size may be necessary to fully develop associations within distinct domains. The sample sizes used in the present study are small given the potential heterogeneity that exists within the epigenetic and symptom data across the participants, therefore, these findings warrant replication and confirmation in larger cohorts. Further, for feasibility of collecting data in settings for military training, there was only limited opportunity available for capturing lifetime history information. More detailed information on not only frequency of blast exposures, but also magnitude of exposures, as well as other factors such as lifetime trauma and military related post-traumatic stress, was not practical to obtain and thus reduces the precision in this work. Finally, although the differentially methylated loci identified link to relevant clinical features related to chronic symptoms of tinnitus, sleep difficulties, and headache reported by the breachers, it is important to demonstrate in future studies whether these transcriptional regulatory changes confer coordinated changes in gene expression *via* whole genome RNA sequencing within the same samples and subjects, thus allowing for establishment of functional association.

## Conclusions

The findings from this study have broad implications, which have led to identification of molecular signatures in military service members that track with chronic symptoms related to cumulative exposure to blast. These molecular signatures are measured through changes in DNA methylation, which is a highly stable epigenetic mark that can encapsulate a lifetime of environmental exposures. DNA methylation alterations in genes identified in this study, which tracked with chronic symptoms of tinnitus, sleep disturbance, and pain prevalent in service members and veterans, provides mechanistic insight on the impact of repeated blast injury and related sequelae, thus, allowing for consideration of prevention measures during training and deployment of our Warfighters, as well as informing future development of biomarkers in clinical care for our veterans.

## Data Availability Statement

The datasets have been uploaded to the GEO (accession #: GSE155426).

## Ethics Statement

The studies involving human participants were reviewed and approved by Institutional Review Board (IRB) of the Walter Reed Army Institute of Research (Silver Spring, MD). The patients/participants provided their written informed consent to participate in this study.

## Author Contributions

AB, CL, GK, and WC contributed to conceptualization of study protocol, recruitment, to phenotypic, and biospecimen collection. FH, YG, CW, and ZW contributed to study-design and conducted experiments. JN inventoried and shipped the samples. CW conducted sample processing and quality control. ZW performed DNA methylation and symptom data analyses with statistical/analytical guidance from YG. CW, ZW, and FH prepared the manuscript. All authors reviewed and approved the manuscript. All authors contributed to the article and approved the submitted version.

## Conflict of Interest

AB is the founder of Aries Biotechnologies, a consulting firm based in Oakland, CA. The remaining authors declare that the research was conducted in the absence of any commercial or financial relationships that could be construed as a potential conflict of interest.

## Supplementary Material

Figure S1 Quality control, detection *p*-value. Bar graph showing the proportion of probes with poor quality (*p* > 0.0005) probes for each biological sample. All samples had high quality data, with at least 99% of probes passing criteria.

Figure S2 Quality control, gender prediction. Plot demonstrates consistency for predicted and reported sex, using Chromosome X and Y median intensity.

Figure S3 Quality control, genotype consistency check with paired samples pre- and post-training exposure. The dendrogram shows clustering of individuals by genotypes derived from the 65 SNP probes for biological samples collected pre/post training. The y-axis shows Euclidean distance and samples that show no clustering were those for which no post-training biological samples were available.
