# Supplementary figures and images for: DNA Methylation Patterns of Chronic Explosive Breaching in U.S. Military Warfighters
Source: Front Neurol. 2020 Oct 23;11:1010. doi: 10.3389/fneur.2020.01010 (PMC7645105; doi:10.3389/fneur.2020.01010)

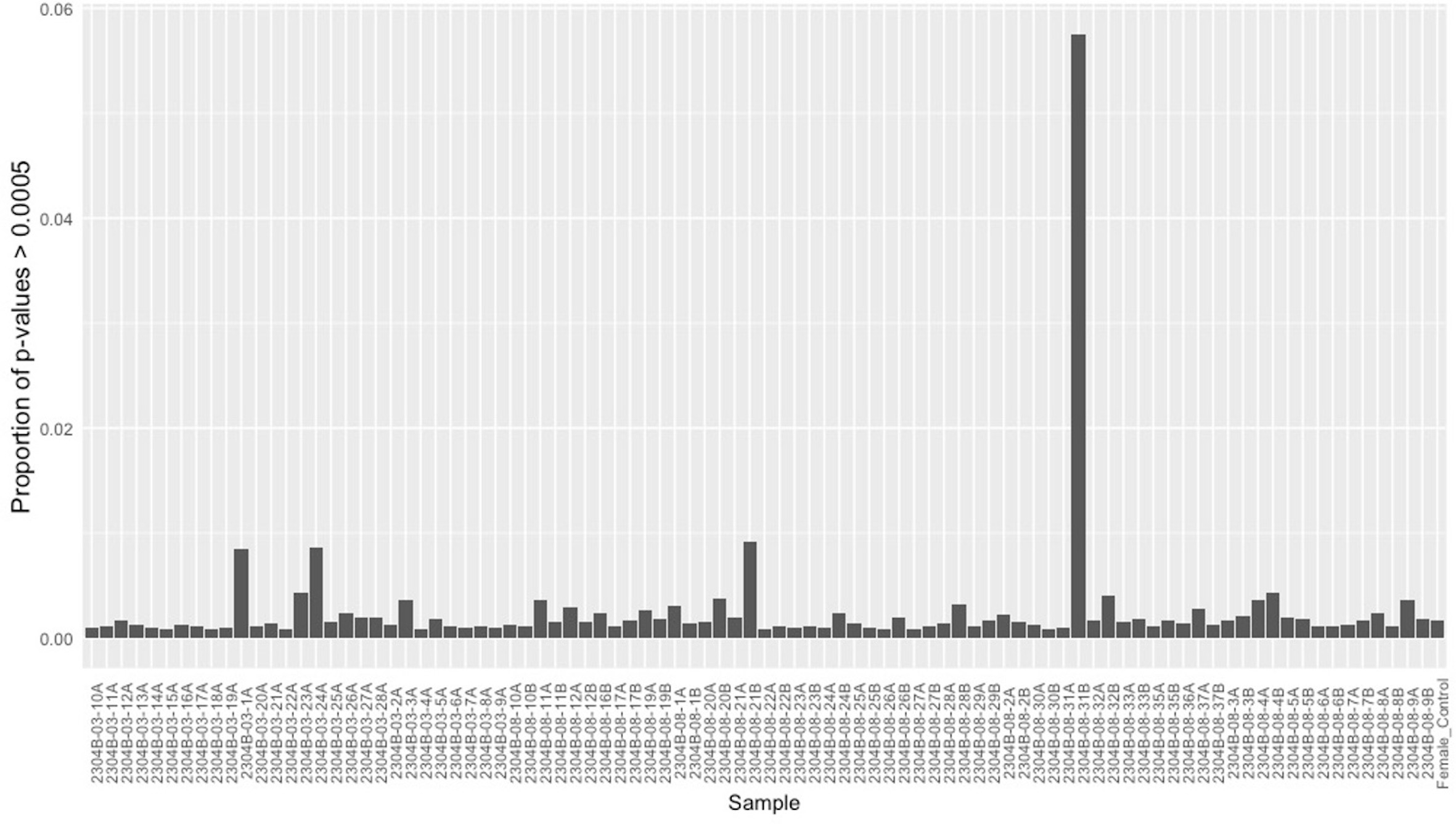

Supplement: Figure S1 — Quality control, detection p-value. Bar graph showing the proportion of probes with poor quality (p > 0.0005) probes for each biological sample. All samples had high quality data, with at least 99% of probes passing criteria. [file Image_1.JPEG]

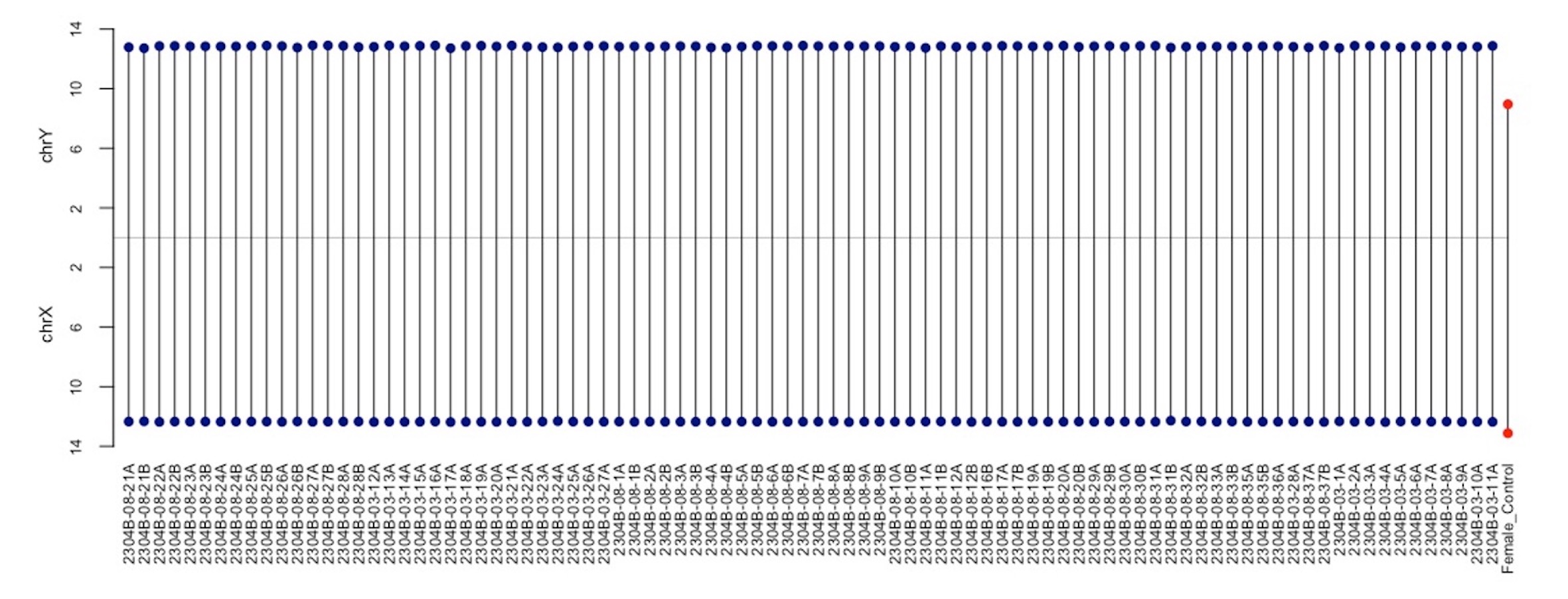

Supplement: Figure S2 — Quality control, gender prediction. Plot demonstrates consistency for predicted and reported sex, using Chromosome X and Y median intensity. [file Image_2.JPEG]

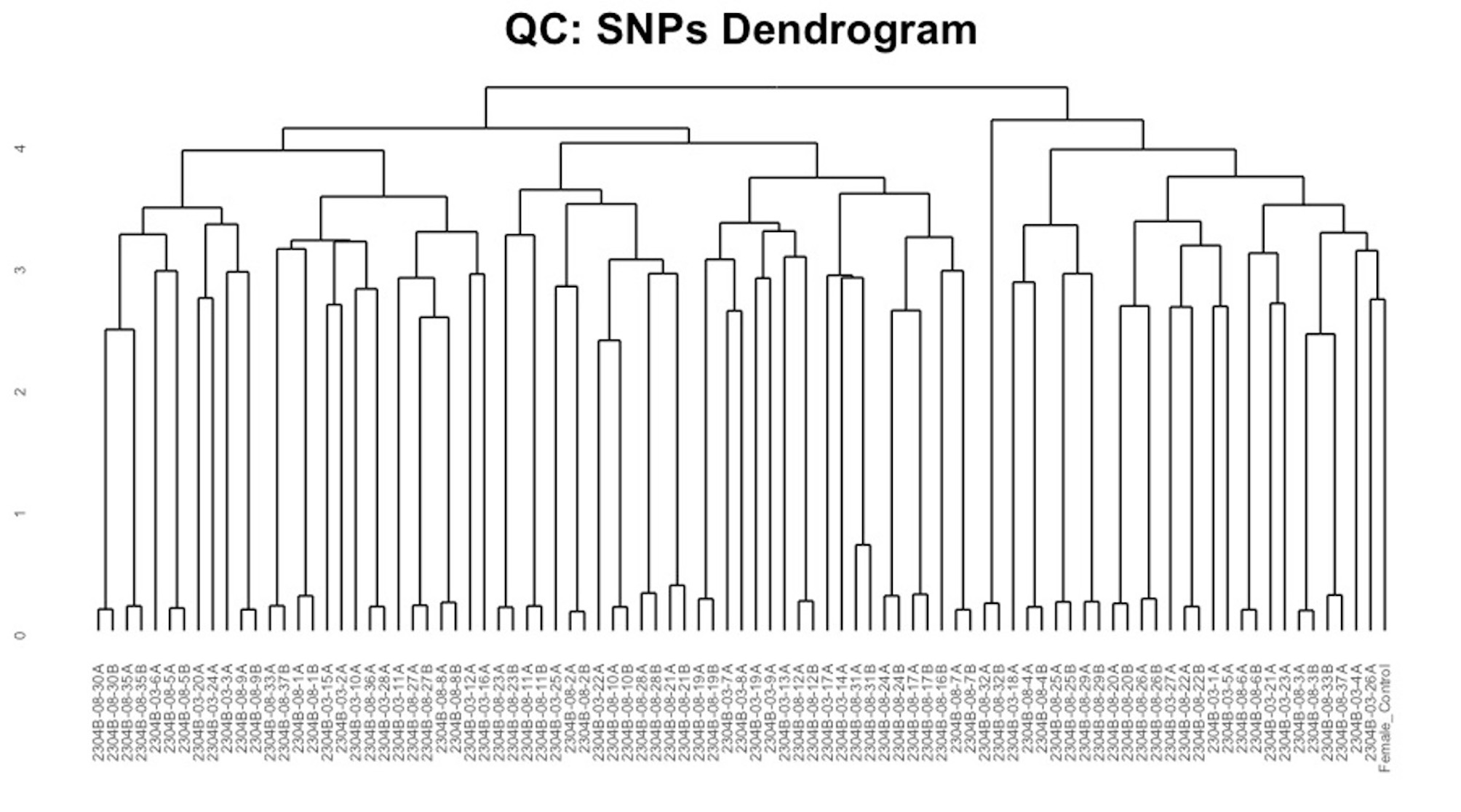

Supplement: Figure S3 — Quality control, genotype consistency check with paired samples pre- and post-training exposure. The dendrogram shows clustering of individuals by genotypes derived from the 65 SNP probes for biological samples collected pre/post training. The y-axis shows Euclidean distance and samples that show no clustering were those for which no post-training biological samples were available. [file Image_3.JPEG]
